# Antimicrobial Resistant *Enteropathogenic Escherichia coli* and *Salmonella* spp. in Houseflies Infesting Fish in Food Markets in Zambia

**DOI:** 10.3390/ijerph14010021

**Published:** 2016-12-28

**Authors:** Mwansa M. Songe, Bernard M. Hang’ombe, Theodore J. D. Knight-Jones, Delia Grace

**Affiliations:** 1 Food Safety and Zoonoses Program, Zambia, International Livestock Research Institute (ILRI), Lusaka 10101, Zambia; tknightjones@gmail.com; 2 Paraclinical Studies Department, School of Veterinary Medicine, University of Zambia, Lusaka 10101, Zambia; mudenda68@yahoo.com; 3 Food Safety and Zoonoses Program, Kenya, International Livestock Research Institute (ILRI), Nairobi 00100, Kenya; D.GRACE@CGIAR.ORG

**Keywords:** fish, informal food markets, houseflies, *Escherichia coli*, *Salmonella*, antimicrobial resistance

## Abstract

Diarrhea is one of the most common diseases and is a leading cause of death in developing countries. This is often caused by contaminated food. Poor food hygiene standards are exacerbated by the presence of flies which can transmit a variety of infectious microorganisms, particularly through animal source foods. This fact becomes especially important in developing countries like Zambia, where fish is a highly valued source of protein. Our interest in this study was to identify if the flies that beset food markets in Zambia carry important pathogenic bacteria on their bodies, and subsequently if these bacteria carry resistance genes to commonly used antibiotics, which would indicate problems in eradicating these pathogens. The present study took into account fish vendors’ and consumers’ perception of flies and interest in interventions to reduce their numbers. We conducted semi-structured interviews with (1) traders (comprised of randomly selected males and females) and (2) consumers (including randomly selected males and females). Thereafter, we collected flies found on fish in markets in Mongu and Lusaka districts of Zambia. For the entire study, a total of 418 fly samples were analyzed in the laboratory and *Salmonella* spp. and enteropathogenic *Escherichia coli* were isolated from the flies. Further laboratory screening revealed that overall, 17.2% (72/418) (95% CI; 43.2%–65.5%) of total samples analyzed contained Extended-Spectrum Beta-Lactamase (ESBL)-producing *E. coli*. These significant findings call for a strengthening of the antibiotic administering policy in Zambia and the development of sustainable interventions to reduce fly numbers in food markets and improve food safety and hygiene.

## 1. Introduction

Enteric and diarrheal diseases are important causes of childhood death in the developing world [1]. These diseases are responsible for more than 750 thousand deaths in children under 5 years old worldwide, ranking them as the second cause of death, after lower respiratory diseases, in this age group [2]. Diarrheal diseases are often caused by contaminated food: the first assessment of the global burden of foodborne disease estimated a burden of 600 million cases of foodborne illness and 420,000 deaths in 2010 [3]. In most developing countries including Zambia, gastro-intestinal disease remains in the top five causes of sickness and death and unsafe food is an important contributor to this avoidable burden [4]. The impact of poor food hygiene on public health due to an increased incidence of gastro-intestinal disease is even further aggravated by the presence of flies that serve as vectors for a variety of pathogenic microorganisms. Many published studies show that flies can carry bacteria, viruses and parasites [5,6,7,8,9,10,11,12]. Particularly in urban areas near human dwellings with a high human population density, flies have conducive conditions to grow and reproduce year-round and can play an important role in cross-contamination between dirty contaminated environments and food sources as they fly from one place to another. Poor sanitary conditions prevail in food markets, creating a potential for higher housefly populations. Additionally, simple pit latrines commonly used in most of the Zambian markets may provide increased access to human excreta for flies, further increasing the potential for fly-borne transmission of disease-causing microorganisms [13].

The contamination of food products with *Salmonella* spp. and enteropathogenic *E. coli* generates serious health and economic consequences. Bacteria from the genus *Salmonella* cause illnesses such as typhoid fever, paratyphoid fever, and food poisoning. *Salmonella* spp. are transmitted primarily through contaminated food and water [14,15].

*E. coli*, a commensal bacterium of humans and animals [15], is a significant cause of gastrointestinal disease, ranging from simple diarrhea to dysentery-like conditions [15]. *E. coli* has also been shown to carry drug resistant genes that may complicate management of diarrheal diseases [16]. *E. coli* is sometimes used as a sentinel for monitoring antimicrobial drug resistance in fecal bacteria because it is found more frequently in a wide range of hosts and acquires resistance easily [17]. Surveillance data show that resistance in *E. coli* is consistently highest for antimicrobial agents that have been in use the longest time in human and veterinary medicine [18].

Although flies are believed to pose serious health threats by transmission of disease-causing microorganisms, researchers and policy makers in Africa lack up-to-date information on potential disease-causing microorganisms carried by flies and their impact on human health. The aim of our study was to identify if the flies that beset food markets in Zambia carry important pathogenic bacteria on their bodies, and subsequently if these bacteria carry resistance genes to commonly used antibiotics, which would indicate problems in eradicating these pathogens. The study also took into account fish vendors’ and consumers’ perception of flies and interest in interventions to reduce their numbers.

## 2. Materials and Methods

### 2.1. Site Selection and Semi-Structured Interviews

**Figure 1:**
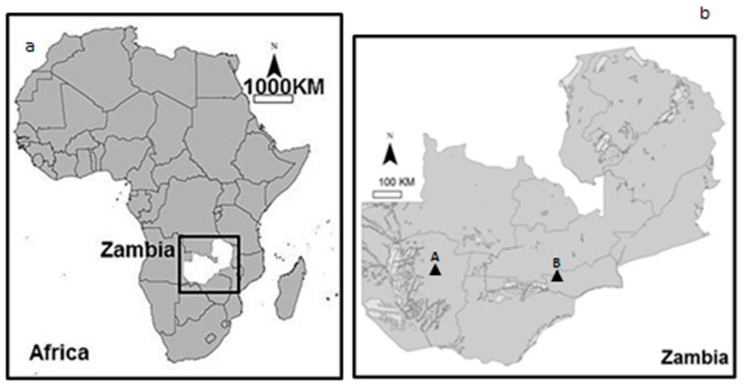
(**a**) Map of Africa showing location of Zambia; (**b**) Map of Zambia showing location of Mongu (A) and Lusaka (B).

This study was conducted in two different markets of Mongu (A) and Lusaka (B) districts (15.2736° S, 23.1501° E and 15.3875° S, 28.3228° E respectively), Zambia (Figure 1). The markets supply a wide range of products from fresh foods to dry foods. Semi-structured interviews were conducted with the traders (comprised of randomly selected males and females) and consumers (including randomly selected males and females), using a simple interview tool, which included the following thematic areas:

(1) How the vendor/consumer feels about the presence of high numbers of flies at fish stalls.
  (a) Whether or not the vendor has tried some control measures against the flies
  (b) The consumer’s view of a fish stall that appears to have some intervention against flies in place.
(2) What interventions those vendors that have had no means of reducing or getting rid of flies would like to try, given a chance.
(3) What the vendors think about covering the fish with netting material to keep flies away from the fish.

The discussions were held with male and female traders and consumers, to understand their perception of flies and interest in interventions to reduce their numbers. Individual males and females that comprised the groups were randomly selected. The survey was conducted in January and April in Mongu and Lusaka respectively. Because consumers were in a hurry to make their purchase and return home, it was easier to have the five-to-ten minute discussion with traders. Ten consumers were interviewed in the Mongu market while 20 traders participated in the interviews. Twenty traders and twenty consumers were interviewed in Lusaka where consumers were more willing to participate in interviews.

### 2.2. Fly Collection and Sampling

Fresh fish was our commodity of interest in this study because fish is one of the most risky foods with regard to food-borne illnesses [4]. Furthermore, fish is a very important high-protein animal-source food consumed by the poor in Zambia. Flies were collected using standard methods as described previously [19]. Fly collection in Mongu took place in March 2015 over a period of four days. In Lusaka flies were collected in June 2015 within a period of two weeks. Individual houseflies found infesting the fish were knocked down, but not squashed, using a fly swatter. After each blow, the fly swatter was treated with 70% ethyl alcohol. The dead flies were retrieved using sterile forceps and deposited into individual 1.8 mL Eppendorf tubes (Zhejiang Medical Technology, Taizhou, China) containing 1 mL phosphate-buffered saline (PBS, HiMedia Laboratories, Mumbai, India). Twenty-five flies were collected on each sampling day, and a total of 418 flies were collected for the entire study. The fly samples were kept in an ice-cooled cooler box and sent to the laboratory within two to three hours of collection. They were frozen upon arrival at the laboratory in the freezer at −20 °C until analysis.

### 2.3. Culture, Isolation and PCR Detection of Salmonella *spp.* and Enteropathogenic E. coli

Before analysis the frozen flies were thawed and the contents of each Eppendorf tube were vortexed for 30 s to initiate exterior washing. After washing, 100 µL of PBS fly wash was subjected to total bacterial and coliform counts on Nutrient agar (Oxoid, Basingstoke, UK) and MacConkey agar (Oxoid, Basingstoke, UK) respectively, using the drop plate methodology, as described before [20]. *Salmonella* spp. and *E. coli* were cultured using the PBS fly wash as the primary inoculum. In the case of *Salmonella* 100 µL of the PBS fly wash was placed in 900 µL selenite broth (Wako Pure Chemical Industries Ltd., Osaka, Japan) for enrichment at 37 °C overnight. The isolation of *Salmonella* spp. was made on Xylose Lysine Deoxycholate agar (HiMedia Laboratories, Mumbai, India) following inoculation and culture for 24 h at 37 °C. The identity of suspected *Salmonella* isolates was confirmed biochemically and serologically by standard methods [21,22]. In the isolation of *E. coli*, the PBS fly wash was inoculated on MacConkey agar and colonies suspected to be *E. coli* through lactose fermenting observation were selected. Identification of *E. coli* lactose-fermenting positive colonies was conducted using phenotypic characteristics and confirmed by the Triple Sugar Iron (TSI) and IMViC tests [21,22].

PCR was used as a further confirmatory test for *Salmonella* spp. and *E. coli*. In order to perform the PCR, the template DNA for *Salmonella* spp. and *E. coli* was extracted by using the heat treatment method as previously described [23,24]. Briefly, the suspected isolates were inoculated in 1 mL of Brain Heart Infusion (BHI) broth (Nissui, Tokyo, Japan), placed in an Eppendorf tube and incubated at 37 °C for 18 to 24 h. After cultivation, 0.5 mL of the BHI broth was subjected to heat treatment on a heat block (Thermo Alumi Bath Alb-220: Iwaki Glass Co. Ltd., Tokyo, Japan) at 95 °C for 10 min. The heated broth was centrifuged at 13,000× *g* for 5 min to obtain the supernatant (DNA sample) which was then stored at −20 °C for further use.

The presence of *Salmonella* spp. was confirmed with the *invA* gene following amplification using previously used primers [25], while Enteropathogenic *E. coli* was confirmed through the detection of the multi-gene region, as previously indicated [23]. The reaction conditions for *E. coli* were 5 μL Phusion^®^ Flash High-Fidelity PCR Master Mix (Thermo Fisher Scientific, Vantaa, Finland), 2 μL distilled water, 2 μL primer mix (MORA-Primer Diarrheal pathogens, Takara Bio Inc., Shiga, Japan), and 1 μL of the DNA sample. Ten microliters reaction mixtures were subjected to amplification for 30 cycles (98 °C denaturing for 10 s and 55 °C for 5 s and 72 °C for 15 s) in PIKO Thermal Cycler (Thermo Fisher Scientific, Vantaa, Finland). Similar conditions were used for *Salmonella* spp. The only difference was that the annealing temperature was at 60 °C.

After PCR, products were directly added to 2 μL of gel loading dye (New England Biolabs, Ipswich, MA, USA) to prepare samples for electrophoresis. Samples were gently applied (2 μL per well) to agarose gel (1% agarose and 1% synergel, Wako Pure Chemical Industries Ltd., Osaka,, Japan). Electrophoresis was done at 100 V until dye markers migrated to an appropriate distance, depending on the size of DNA to be visualized. A 100 to 1517 bp DNA ladder moleculer weight marker (Quick-Load^®^ 100 bp DNA Ladder, New England Biolabs, Ipswich, MA, USA) was used to identify the corresponding amplified products. Gels were stained with ethidium bromide and observed under ultraviolet illumination at 256 nm. *Salmonella* strains previously identified originating from chicken carcasses were used as positive controls while *E. coli* was used as a negative control. In case of *E. coli*, EDL931 (stx-1/stx-2 positive strain) was used as a positive control and the known *Salmonella* as a negative control.

### 2.4. Determination of Extended Spectrum Beta-Lactamase (ESBL) Producing E. coli and Antibiotic Susceptibility Patterns

The detected enteropathogenic *E. coli* were inoculated on MacConkey agar (Oxoid, Basingstoke, UK) containing 2 mg/L of cefotaxime (Sigma-Aldrich, Munich, Germany) for preliminary screening of ESBL-producing bacteria [23,25]. The plates were later incubated at 37 °C for 24 h and the colonies that grew on MacConkey agar were subjected to genetic determination of ESBL producing. The *E coli* isolates were cultured on brain-heart-infusion followed by DNA extraction using the heat treatment method. The *E. coli* isolates were then subjected to PCR for confirmation of resistance genes TEM (Temoniera), SHV (Sulphydryl Variable) and CTX-M (Cefotaxime–Munich) using primers previously used by other workers [21,26,27,28]. The PCR (Finnzymes Oy, Espoo, Finland) was performed in a total reaction volume of 10 µL consisting of 5 µL Phusion master mix, 2 µL sterile distilled water, 2 µL primers (forward and reverse) and 1 µL bacterial DNA template. The PCR was performed using the rapid cycle DNA amplification method comprising of an initial denaturation step at 98 °C for 30 s, followed by 35 cycles of template denaturation at 98 °C for 1 s, primer annealing at 60 °C for 5 s and 72 °C for 1 s with final extension at 72 °C for 10 s. The PCR products were later viewed with ethidium bromide after electrophoresis through 1.5% agarose gel.

The antimicrobial susceptibility testing was done using the Kirby-Bauer disc diffusion method on Mueller Hinton Agar (Becton, Dickinson and Company, Sparks, MD, USA) based on the Clinical Laboratory Standard Institute (CLSI) guidelines [24]. The antibiotic discs (Becton, Dickinson and Company, Sparks, MD, USA) used included ampicillin (10 μg), sulfamethoxazole/trimethoprim (1.25/23.75 μg), streptomycin (300 μg), ciprofloxacin (5 μg), tetracycline (30 μg), gentamicin (10 μg), nalidixic acid (30 μg), chloramphenicol (30 μg), ceftazidime (30 μg), norfloxacin (10 μg) and cefotaxime (30 μg). The phenotypic confirmation of ESBL isolates was done by the combination of disc approximation method using either ceftazidime (30 μg) or cefotaxime (30 μg) alone followed by over-night incubation at 37 °C for 18 to 24 h. Interpretation of susceptibility patterns on other anti-microbial discs was done using guidelines laid down in the CLSI, which provides break points corresponding to zones of inhibition diameter. Quality control standard laboratory procedures were strictly followed to avoid contamination. *Escherichia coli* ATCC 25922 was used as a quality control organism.

## 3. Results

### 3.1. Semi-Structured Interviews

All 20 consumers in Lusaka and 10 consumers in Mongu said they would prefer to buy fish from a trader that employed an intervention, such as the use of chlorinated water to disinfect the fish stalls, which could help reduce the number of flies infesting the fish. One consumer stated, “*When I see a lot of flies infesting the fish, I get put off and decide not to purchase from that particular vendor. I have noticed that those vendors who make an effort to disinfect their stalls have fewer flies on their stall and that is very appealing*”. However, four of the ten consumers in Mongu (40%) pointed out that a complete absence of flies might mean that the trader had treated their fish with chemicals that reduce flies but could be harmful to humans and hence an absence of flies might be a deterrent. All twenty traders in both markets complained that flies shorten the shelf-life of their fish and their presence in high numbers at the stall gives the impression that the fish is unhygienic, discouraging potential customers from purchasing the commodity. All the traders spoken to in the Lusaka market requested for help in getting rid of the flies, as they are aware of the role they play as disease-causing agents but do not know have the means to get rid of them or at least reduce their numbers. Two out of the twenty (10%) traders interviewed in the Mongu market, when asked what they thought about the use of nets to keep flies away, said they did not like the idea Their reason for this was that the presence of nets on their stall would deter potential customers from buying fish from them. The rest of the traders in both markets liked the idea of using nets, as long as there was a neat way of placing them over the fish.

### 3.2. Salmonella *spp.* and E. coli in Flies

Fly contamination was determined through the growth of *E. coli* and *Salmonella* spp. A total of 418 flies were examined in this study. Of these flies 100 were caught in Mongu and the rest in Lusaka. In Lusaka 221 out of 318 flies (69%) were found contaminated with *E. coli*, while 86 of the 100 flies (86%) caught in Mongu were positive for *E. coli*. In the case of *Salmonella* spp., 23 of the 318 flies (7.2%) from Lusaka were positive while only 6 flies (6%) were positive from Mongu samples. *Salmonella* spp. were detected through the presence of the *invA* gene on PCR.

An attempt to detect the virulence of pathogenic *E. coli* was made. The virulent genes belonging to enteroaggregative *E. coli* (EAST 1), enterohemorrhagic *E. coli* (stx-1) and enterohemorrhagic *E. coli* (stx-2) were detected. The most frequently detected gene was the EAST 1 gene in flies from both Lusaka and Mongu, while recordings for stx-1 and stx-2 were relatively low for both the Lusaka and Mongu markets. Table 1 below summarizes the number of positives/number tested for each market.

### 3.3. ESBL and Antimicrobial Susceptibility Patterns of Salmonella *spp.* and E. coli

Overall, 56 ESBL-producing *E. coli* isolates were obtained from flies in Lusaka representing 25% (56/221 of flies positive for *E. coli*) while none were observed in the 86 flies obtained from Mongu. In the case of *Salmonella* spp., all the isolates (23 isolated from Lusaka and 6 from Mongu) were susceptible to the tested antibiotics. Of these ESBL producing *E. coli*, 42 isolates carried *bla*_CTX-M_ genes. Of the 42 *bla*_CTX-M_ positive isolates, 18 (42.9%) isolates were positive for *bla*_CTX-M_ genes and 15 (35.7%) were positive for *bla*_SHV_ genes. Some isolates (21.4%), were positive for both *bla*_TEM_ and *bla*_SHV_ genes simultaneously. See Table 2 for a summary.

All the 42 *bla*_CTX-M_ positive isolates were subjected to antimicrobial susceptibility tests where drug-resistant phenotypes were observed. All the 42 isolates (100%) were resistant to cefotaxime and ampicillin, while 40 out of 42 isolates 95.2%) were resistant to ceftazidime and ciprofloxacin. The two out of 42 isolates (4.8%) susceptible to ceftazidime and ciprofloxacin, were observed not to possess the *bla*_SHV_ genes. Eleven of the 42 *bla*_CTX-M_ isolates (26.2%) were resistant to all the 11 antibiotics, while 40 out of 42 (95%) *bla*_CTX-M_ positive isolates were resistant to at least 6 antibiotics. The highest resistance among non-beta-lactam antibiotics was found to be against nalidixic acid (93.0%), followed by tetracycline, norfloxacin and gentamycin (91.0% each), streptomycin (88.1%), chloramphenicol (81.0%) and Sulfamethoxazole-trimethoprim (74.0%). See Table 3.

## 4. Discussion

The lack of information on antimicrobial resistance in sub Saharan Africa is a major problem, particularly considering the limited control of antibiotic usage in many countries in the region. Therefore, findings from this study are significant as they present the first report on detection of *Salmonella* spp. and antimicrobial resistant enteropathogenic *E. coli* in flies infesting fresh fish in market places in Zambia. For the fish retailer as well as consumer, flies are a nuisance which is accepted as part of everyday life. Flies from environments contaminated with human pathogens readily become contaminated themselves [19]. This phenomenon has been shown through numerous studies by different researchers [29,30,31,32,33]. The finding of *Salmonella* spp. is significant in that salmonellosis is one of the most widespread food-borne zoonoses in the world, and the problem is increasing both in industrialized and developing countries. *Salmonella* spp. are capable of infecting both humans and animals and are one of the major causes of diarrheal diseases all over the world [24].

*E. coli* has the capability of acquiring and preserving transferable resistance genes found in other organisms and the environment. In addition, this organism is also an important pathogen causing a variety of illnesses, including urinary and gastrointestinal infections and septicaemia [34]. The major mechanisms of resistance to oxyimino-cephalosporins in *E. coli* rely on the production of extended-spectrum-lactamases [35]. The fact that ESBL-producing *E. coli* isolates were obtained from 25% of the flies in Lusaka gives an indication of the presence of anti-microbial resistant gene-carrying *E. coli* in the communities. It is important to mention that the study site in Lusaka is a cosmopolitan market that handles foreign and local traders dealing in many forms of merchandise ranging from vegetables and livestock to various other foodstuffs. The market in Mongu is in a much more rural setting with fewer merchandise items. Resistant infections lead to increased morbidity and prolonged hospital stays, as well as to prolonged periods during which individuals are infectious and can spread their infections to other individuals [36,37]). The problem is particularly severe in developing countries, where the burden of infectious diseases is relatively greater and where patients with a resistant infection are less likely to have access to or be able to afford expensive second-line treatments, which typically have more complex regimens than first-line drugs. Furthermore, the presence of exacerbating factors, such as poor hygiene, unreliable water supplies and increased numbers of immunocompromised patients can further increase the burden of antimicrobial resistance by facilitating the spread of resistant pathogens [38].

Our findings in this study further justify the semi-structured interviews respondents’ concern over the poor sanitary conditions and lack of formal refuse collection facilities which would serve as breeding grounds for disease-causing organisms in the markets. Flies have long been associated with the potential for spreading disease because of their intimate relationship with decaying matter, garbage, and feces [19].

The use of participatory methods (involvement of retailers and consumers through semi-structured interviews) gave the researchers a quick overview of stakeholders’ perceptions of flies. Most of the participants of this participatory process were able to communicate their need for an intervention that could help reduce the fly populations in the market places. Prior engagement with the market agents from the onset made the fly collection process, which would have otherwise been a socially awkward process, a lot easier. Note should be taken, however, that for some positive changes to occur, key decision-makers should be involved in the process as they are the ones who could ultimately improve sanitary conditions in market places.

It is important to recognize that although this study focused on flies collected from fish, this commodity is usually cooked before it is eaten, and so any enterobacteriaceae will die during these procedures. Our concern, though, is cross contamination of other products such as fresh salad, fruit and groundnuts with which the fish is in close proximity, as well as the indication of high pathogen prevalence and antimicrobial resistance contamination of environment and foods.

## 5. Conclusions

The high levels of contamination with *Salmonella* spp. and antimicrobial resistant enteropathogenic *E. coli* detected in flies in this study are an indication of a serious threat to animal and public health. Also, flies are a menace to fish traders, whose fish selling business is negatively affected by high fly numbers. Both fish traders and consumers would greatly appreciate an intervention, such as the use of nets, against flies at fish stalls as a practical way of addressing the underlying causes of compromised food safety. A follow-up pilot study to investigate whether or not the use of nets to keep flies away from fish stalls would lead to a significant reduction of microbial load on fish was conducted. Results will be presented in a separate publication.

## Figures and Tables

**Table 1:** Percentage of *E. coli* positive for EAST 1, stx-1 and stx-2 virulent genes for both markets.

Total Number of Flies Positive for *E. coli*	EAST + ve	stx-1 + ve (%)	stx-2 + ve (%)
Lusaka (*n* = 221)	72/221 = 32.6%	12/221 = 5.4%	23/221 = 10.4%
Mongu (*n* = 86)	26/86 = 30.2%	8/86 = 9.3%	6/86 = 7.0%

**Table 2:** *bla*_TEM_ and *bla*_SHV_ genes in *bla*_CTX-M_ positive isolates.

Gene	Number of Isolates	Percentage (%)
*bla*_TEM_	18	42.9
*bla*_SHV_	15	35.7
*bla*_TEM_ and *bla*_SHV_	9	21.4
Total	42	100

**Table 3:** Pattern of antimicrobial resistance in *bla*_CTX-M_ producing *E. coli* isolates.

Antibiotic	No. of Isolates Showing Resistance	Percent (%)
Ampicillin (AMP)	42	100
Sulfamethoxazole-trimethoprim (SXT)	31	74.0
Streptomycin (STR)	37	88.1
Tetracycline (TET)	38	91.0
Gentamycin (GEN)	38	91.0
Nalidixic acid (NAL)	39	93.0
Ceftazidime (CAZ)	40	95.2
Chloramphenicol (CHL)	34	81.0
Norfloxacin (NOR)	38	91.0
Ciprofloxacin (CIP)	40	95.2
Cefotaxime (CTX)	42	100
